# Supplementary material for: qByte: An open-source isothermal fluorimeter for democratizing analysis of nucleic acids, proteins and cells
Source: PLoS Biol. 2025 May 28;23(5):e3003199. doi: 10.1371/journal.pbio.3003199 (PMC12157786; doi:10.1371/journal.pbio.3003199)
Supplement: Attachment — Submitted filename: qByte review 2025 - Saad bhamla.pdf [file pbio.3003199.s003.pdf]

The authors have demonstrated how to build an open-source fluorimeter using digital manufacturing and standard Printed Circuit Boards (PCB) assembly and benchmarked it against commercial instruments, including real-time 46 thermocyclers and isothermal spectrophotometers, showing comparable results while offering lower production costs and greater accessibility. They have validated it in various real-world scenarios, demonstrating its portability and reliability in the environmental detection of human pathogens, characterizing enzyme activities, and studying bacterial viability. Overall it's a nice study and does solve an existing problem that this reviewer was engineering so now we can pause our project and build on this team's open-source hardware. It's good to be scooped in this way. We do have a few comments that if the authors can address, will make the paper stronger.

There are a few other similar fluorimeter hardware designs — how do they compare with what the authors have done? A table (perhaps in SI) with detailed comparison/benchmarking with the author's paper will help future readers/builders understand how this design fares compared to other existing design. Perhaps a short para in discussion (or in SI) would be useful.

Hardware Refs:

1. Combination-Lock SlipChip Integrating Nucleic Acid Sample Preparation and Isothermal LAMP Amplification for the Detection of SARS-CoV-2, Jiajie Zhang, Lei Xu, Zheyi Sheng, Jiayi Zheng, Weiyu Chen, Qixin Hu, and Feng Shen, ACS Sensors 2024 9 (2), 646-653, DOI: 10.1021/acssensors.3c01727
(addresses sample preparation and amplification)

2. Low-Cost, Open-Source Device for High-Performance Fluorescence Detection of Isothermal Nucleic Acid Amplification Reactions, Andrew H. Buultjens, Koen Vandelannoote, Liam K. Sharkey, Benjamin P. Howden, Ian R. Monk, Jean Y. H. Lee, and Timothy P. Stinear, ACS Biomaterials Science & Engineering 2021 7 (10), 4982-4990, DOI: 10.1021/acsbiomaterials.1c01105

3. García-Bernalt Diego J, Fernández-Soto P, Márquez-Sánchez S, Santos Santos D, Febrer-Sendra B, Crego-Vicente B, Muñoz-Bellido JL, Belhassen-García M, Corchado Rodríguez JM, Muro A. SMART-LAMP: A Smartphone-Operated Handheld Device for Real-Time Colorimetric Point-of-Care Diagnosis of Infectious Diseases via Loop-Mediated Isothermal Amplification. Biosensors (Basel). 2022 Jun 16;12(6):424. doi: 10.3390/bios12060424. PMID: 35735571; PMCID: PMC9221248.

Major Comments

- The problem the authors want to solve is building a standalone, production-ready, and affordable scientific equipment for a wide range of biological applications. However, they have not critically addressed the bottlenecks in the workflow, i.e., sample preparation in the field, which can make or break the assay/reaction and requirements of ancillary devices to purify the samples. The field samples that were conducted already used purified samples that were independently procured; Can the authors comment on this?

- I suggest that the authors include a discussion for sample preparation and ancillary equipment in either their results or discussion sections as advancements in isothermal nucleic acid (NA) amplification have shifted the bottleneck in point-of-care (POC) nucleic acid amplification tests (NAATs) to sample preparation. Crude samples often contain inhibitors that affect polymerase activity or fluorescence detection, as seen in SARS-CoV-2 RT-PCR tests, where components of viral transport media can inhibit PCR reaction. This makes NA sample preparation essential before testing. Unlike amplification, sample preparation is a multi-step separation process to isolate NAs from lipids, proteins, and polysaccharides. Challenges vary by specimen type, including blood, swabs, saliva, sputum and urine. Ultimately, it is as critical to the NAAT workflow as amplification itself. [S. Soni *et al.* Paper-based nucleic acid sample preparation for point-of-care diagnostics, Sensors Actuators B Chem., (2022)] Even if not addressed in this paper - perhaps this would serve

as future work for the next team that picks up the baton and builds on this qbyte platform.

- The LED driver drives 8 LEDs simultaneously, which means if there are differences in intensity in LEDs, they will likely lead to errors in measurement. Here, no absolute intensity measurements are done as acknowledged by the authors; this will likely lead to errors in the field during its lifetime. So, software-based calibration steps could have been incorporated into the protocol using reference dyes before the tests began to normalize the data.

- The precision of temperature control required for detecting SNPs and other genetic variations is critical, and generally, 0.1^{o}C temperature resolution or better is recommended. Also, errors in temperature measurements at higher temperatures are likely, so calibration curves for the temperature inside the tube measured with a reference thermocouple and temperature measured with the thermistors should have been done at higher temperatures (around denaturation, which is essential for Melt Curve analysis). The peak-to-peak distance while SNP detection can be very close (0.3-0.5^{o}C approx), which is reported in studies like
    - (Harrison LB, Hanson ND. High-Resolution Melting Analysis for Rapid Detection of Sequence Type 131 Escherichia coli. Antimicrob Agents Chemother. 2017 May 24;61(6):e00265-17. doi: 10.1128/AAC.00265-17. PMID: 28416542; PMCID: PMC5444143.)
    - Bruzzone, C.M., Steer, C.J. (2015). High-Resolution Melting Analysis of Single Nucleotide Polymorphisms. In: Bugert, P. (eds) Molecular Typing of Blood Cell Antigens. Methods in Molecular Biology, vol 1310. Humana Press, New York, NY. https://doi.org/10.1007/978-1-4939-2690-9_2
- So, accounting for errors in temperature measurements and temperature resolution is crucial for High-resolution melt curve analysis. However, the authors acknowledge this trade-off between affordability and precision, noting that using cost-effective components and manufacturing methods introduces a higher error level compared to high-end instruments. They suggest potential improvements, including enhancing the optical subsystem for better absolute fluorescence comparison and integrating an additional light channel for ratiometric fluorescence measurements or absorbance readings. Such features could improve the device's accuracy and broaden its applicability in various experimental contexts.

- The authors have done a great job at standardizing the protocols for the four applications they mentioned and have demonstrated satisfactory results for each. Also, they have made significant efforts in designing and implementing Open Source Hardware philosophy in developing this device and have given clear instructions to the end user of each step required for manufacturing and assembly of the device. The software protocol is also clearly established to ensure ease of use for the end user. I commend the team on this effort. Kudos!
- **Filter Selection and Fluorescent Dyes**: Here, commercial LLE filters are used, but for other dyes, it isn't easy to find satisfactory performance using these plastic filters.

    - **Background Fluorescence Interference**: Plastic filters can exhibit inherent fluorescence, leading to background signals that interfere with detecting target fluorescence. A study on reported that conventional color filters, especially those made from certain plastics, can contribute to auto-fluorescence, thereby compromising the sensitivity of fluorescence measurements.
    Yamazaki, M., Krishnadasan, S., deMello, A. J., & deMello, J. C. (2012). Non-emissive plastic colour filters for fluorescence detection. Lab on a Chip, 12(21), 4313–4320. https://doi.org/10.1039/C2LC40718C

-

- **Material Stability and Degradation**: Plastic filters may degrade over time, especially under prolonged exposure to light used in fluorometric assays. This degradation can alter their optical properties, affecting the accuracy and reliability of fluorescence measurements.

Espí, E., Salmerón, A., & García, J. (2006). Plastic films for agricultural applications. Polymer-Plastics Technology and Engineering, 45(8), 875–883. https://doi.org/10.1177/8756087906064220

- Also, volume variation (well to well) calibration has not been investigated, which can lead to major differences in intensity and lead to errors in Melt curve analysis. This is somewhat mentioned in the discussion section but needs further clarification.
- Related to previous comment can the authors show sample sizes ranging from 8 uL to 50 uL? This is relevant to many of the applications the authors demonstrate.

Minor Comments

- Small changes:
  - should use thermal instead of thermic; thermic represents energy in general, as per Merriam Webster; thermal should be limited to heat. (line number:69)
  - It reaches the photodiode. (line number:94)
- Should mention the wattage of resistors used; otherwise leads to the deterioration of resistors over time and their lifetime. (line number:106)
- **Data standardization and interoperability**: To enhance data standardization and interoperability for the qByte device, implementing the Real-Time PCR Data Markup Language (RDML) is recommended.
