# Supplementary material for: qByte: An open-source isothermal fluorimeter for democratizing analysis of nucleic acids, proteins and cells
Source: PLoS Biol. 2025 May 28;23(5):e3003199. doi: 10.1371/journal.pbio.3003199 (PMC12157786; doi:10.1371/journal.pbio.3003199)
Supplement: Attachment — Submitted filename: Response to reviewers.pdf [file pbio.3003199.s004.pdf]

Dear Editor,

Please find below our detailed response intercalated (in blue) with the reference to the main text modifications (*in purple*), fully addressing all the reviewers' comments and suggestions.

Reviewer 1

The authors have demonstrated how to build an open-source fluorimeter using digital manufacturing and standard Printed Circuit Boards (PCB) assembly and benchmarked it against commercial instruments, including real-time thermocyclers and isothermal spectrophotometers, showing comparable results while offering lower production costs and greater accessibility. They have validated it in various real-world scenarios, demonstrating its portability and reliability in the environmental detection of human pathogens, characterizing enzyme activities, and studying bacterial viability. Overall it's a nice study and does solve an existing problem that this reviewer was engineering so now we can pause our project and build on this team's open-source hardware. It's good to be scooped in this way. We do have a few comments that if the authors can address, will make the paper stronger.

We thank the reviewer for his appraisal of our project and its relevance. Indeed, our project was available online all through its development, we thus regret we were not able to join forces earlier. The essence of open hardware is the ability to continue and improve and diversify qByte and we look forward to seeing such contributions and innovations in the future.

There are a few other similar fluorimeter hardware designs — how do they compare with what the authors have done? A table (perhaps in SI) with detailed comparison/benchmarking with the author's paper will help future readers/builders understand how this design fares compared to other existing design. Perhaps a short para in discussion (or in SI) would be useful.

We referred in Lines 30-34 of the original manuscript the myriad of options available. We now added to the Discussion (Lines 333-347) the following phrases and included the requested Table in the supplementary Information(SD1):

Taken together, qByte remains an exceptionally low-cost and versatile tool for real-time assays. Compared to other open-source devices—including Miriam, Snodgrass Device, SMART-LAMP, Buultjens Device, and commercial solutions, as summarized in Table 1 of SD1—qByte offers the most affordable alternative that is production-ready and has been extensively validated, demonstrating replicable results across diverse applications requiring precise dynamic temperature control and fluorescence readouts.

A key distinction of this work from previous ones is the extensive validation of temperature uniformity. It is essential in quantitative applications to ensure that variations in reaction outcomes are not mistakenly attributed to differences in sample concentration or composition when, in reality, they are caused by temperature fluctuations. To achieve this, we employed not only the internal system sensors but we also validated them employing melting probes translating the actual reaction temperature into a fluorescent signal (SD1C). To demonstrate the robustness of this approach, we conducted comparative analyses across replicates in every application, confirming reliable consistency between qByte and commercial solutions,

Hardware Refs:

1. Combination-Lock SlipChip Integrating Nucleic Acid Sample Preparation and Isothermal LAMP Amplification for the Detection of SARS-CoV-2, Jiajie Zhang, Lei Xu, Zheyi Sheng, Jiayi Zheng, Weiyu Chen, Qixin Hu, and Feng Shen, ACS Sensors 2024 9 (2), 646-653, DOI: 10.1021/ acssensors.3c01727

   (addresses sample preparation and amplification)

2. Low-Cost, Open-Source Device for High-Performance Fluorescence Detection of Isothermal Nucleic Acid Amplification Reactions, Andrew H. Buultjens, Koen Vandelannoote, Liam K. Sharkey, Benjamin P. Howden, Ian R. Monk, Jean Y. H. Lee, and Timothy P. Stinear, ACS Biomaterials Science & Engineering 2021 7 (10), 4982-4990, DOI: 10.1021/acsbiomaterials.1c01105
3. García-Bernalt Diego J, Fernández-Soto P, Márquez-Sánchez S, Santos Santos D, Febrer-Sendra B, Crego-Vicente B, Muñoz-Bellido JL, Belhassen-García M, Corchado Rodríguez JM, Muro A. SMART-LAMP: A Smartphone-Operated Handheld Device for Real-Time Colorimetric Point-of-Care Diagnosis of Infectious Diseases via Loop-Mediated Isothermal Amplification. Biosensors (Basel). 2022 Jun 16;12(6):424. doi: 10.3390/bios12060424. PMID: 35735571; PMCID: PMC9221248.

We thank the reviewer for these relevant papers. Paper (2) was referenced on line 38 (reference 18), papers 1 and 3 have been incorporated into the main text into lines 329 of the discussion and 194 of "Application 1: Nucleic Acid Tests" (and now are references 43 and 29).

Major Comments

- The problem the authors want to solve is building a standalone, production-ready, and affordable scientific equipment for a wide range of biological applications. However, they have not critically addressed the bottlenecks in the workflow, i.e., sample preparation in the field, which can make or break the assay/reaction and requirements of ancillary devices to purify the samples. The field samples that were conducted already used purified samples that were independently procured; Can the authors comment on this?

  Indeed, our work is focused on achieving a high-quality affordable open hardware. The challenges of sample preparation are inherent and specific to every target and could not be addressed in depth here. Our hope is that by adopting our machine widely, many researchers could now have an affordable manner to optimize (and share!) sample preparation protocols for their target of choice. To this end, we added the following phrase to the discussion (Lines 320-332):

  For example, one of the main bottlenecks in nucleic acid amplification is not the amplification itself but the preceding steps—particularly sample preparation. Many challenges arise from difficulties in accessing DNA (e.g., from complex environmental samples such as soil or water) or the presence of inhibitory compounds carried over during DNA extraction, which can affect reaction efficiency. The protocols used in this work, adapted from previous studies [30, 31], represent a starting point and could be further refined through iterative improvements and open collaborations to help address these critical limitations in the field. Although previous studies have made

progress on this topic [43], its specificity to each application makes it beyond the scope of this paper. Nevertheless, we believe that the widespread availability of qByte would provide researchers with a low-cost platform to optimize detection protocols for specific targets.

I suggest that the authors include a discussion for sample preparation and ancillary equipment in either their results or discussion sections as advancements in isothermal nucleic acid (NA) amplification have shifted the bottleneck in point-of-care (POC) nucleic acid amplification tests (NAATs) to sample preparation. Crude samples often contain inhibitors that affect polymerase activity or fluorescence detection, as seen in SARS-CoV-2 RT-PCR tests, where components of viral transport media can inhibit PCR reaction. This makes NA sample preparation essential before testing. Unlike amplification, sample preparation is a multi-step separation process to isolate NAs from lipids, proteins, and polysaccharides. Challenges vary by specimen type, including blood, swabs, saliva, sputum and urine. Ultimately, it is as critical to the NAAT workflow as amplification itself. [S. Soni *et al.* Paper-based nucleic acid sample preparation for point-of-care diagnostics, Sensors Actuators B Chem., (2022)] Even if not addressed in this paper - perhaps this would serve as future work for the next team that picks up the baton and builds on this qbyte platform.

We agree with the reviewer, as highlighted in our response above that covers much of the same concern: sample preparation, especially for NA detection is a major concern. We note however (see Line 182-183) that the LAMP is inherently more resistant to inhibition, which is one of the reasons for focusing on isothermal amplification (refs 26). We wish also to highlight that qByte is not restricted to NA detection but, as a standalone portable low-cost real-time temperature-controlled fluorimeter it could serve for a plethora of applications, some highlighted in this work (e.g., SNP analysis, fluorescent protein measurement) and others that may be experimented by future research (e.g., fluorophore-mediated water quality tests for heavy metals etc.).

The LED driver drives 8 LEDs simultaneously, which means if there are differences in intensity in LEDs, they will likely lead to errors in measurement. Here, no absolute intensity measurements are done as acknowledged by the authors; this will likely lead to errors in the field during its lifetime. So, software-based calibration steps could have been incorporated into the protocol using reference dyes before the tests began to normalize the data.

We apologize for the lack of clarity. Each LED can be controlled and calibrated individually. The software includes a still-in-development tab for this purpose, which we have begun testing. However, we found that calibration is non-trivial and unnecessary for the applications showcased in this study, placing it beyond the scope of this paper. We show in supplementary materials fluorescence calibration with several different photodiodes under a range of FAM concentrations, negative and positive LAMP reactions and GFP production in cell free. A simple calibration using reference samples to adjust the intercept and gain of each channel does not fully account for the non-linear complexities of the optical path.

1. *There are multiple variables that interact non-linearly:*
    - *Small differences in the tube fittings and holes of the 3D-printed metal holder.*

- *Variability in the positioning of photodiodes and LEDs during SMD soldering.*
- *Differences in the plastic tubes used and their positioning.*
- *Dynamic changes in sample turbidity during reactions (e.g., caused by LAMP amplicons or lysate reactions degradation), which a simple fluorescence measurement misinterprets as a decrease in signal.*
- *Inherent variability in electrical components, where cost and acceptable variance must be balanced across units.*

2. *These factors do not interact linearly. For example:*
   - *A photodiode slightly displaced due to soldering affects the received intensity depending on the angle and the square of the increased/decreased distance.*
   - *Tube positioning can either amplify or counteract these errors non-linearly (There are multiple factors as the tube plastic can act either as a lens or scatter the light depending on the situation), depending on its insertion angle, leading to improved or worsened signal detection.*
   - *A slight difference in the LED's angle when it gets soldered affects the signal in a different way depending on how the errors on the other mentioned variables are—sometimes mitigating the issue if errors in angles are aligned, sometimes exacerbating it if they are opposing.*

Better calibration protocols could indeed help mitigate these issues, but they are not trivial. For example, we thought to use melting probes as by heating and cooling them across their full melting range the device can calibrate a broad fluorescence spectrum (depending on the probe's melting state). This method could be automated by adding the same probe solution in all eight sample channels:

- *First, the device aligns melting peaks to coincide at the same temperature (x-axis calibration, adjusting the temperature model). This melting peak has been previously measured with a commercially available thermocycler.*
- *Once the peaks are aligned, the fluorescence model is adjusted to match absolute fluorescence intensities across channels (y-axis calibration).*

Taken together, we believe this is beyond the scope of this paper. For the applications tested here, the fluorescence result provides correct kinetic curves of the reaction which, when normalized by any of the methods we added, yields the same results as commercial thermocyclers and allows, for example, the correct quantification of initial DNA ranges in the sample (Fig. 2a). However, better calibration would enable obtaining precise absolute values, making DNA quantification with qByte possible, similar to other fluorimetry-based quantification methods.

The discussion was amended in order to summarize the above considerations (Lines 354-372) that now reads:

In the current design, qByte tracks relative fluorescence changes rather than absolute intensities across its eight channels, which sufficiently supports our demonstrated applications that rely on monitoring signal dynamics over time. However, achieving precise absolute comparisons would be valuable for quantifying absolute concentrations of cells, DNA, or proteins using fluorometric assays but would require more advanced calibration protocols. As partially explored in Supplementary Data (where we use reference FAM concentrations), these complexities extend beyond a simple intercept or gain correction: minor variations in electrical component performance, metal holder manufacturing, LED and photodiode positioning, and plastic tube properties can significantly affect the intensity of received light, overshadowing absolute signal measurements. One approach to improving calibration without relying on manual fine-tuning is to use previously measured melting probes (see application 1: Nucleic Acid Tests) to align fluorescence peaks across a known temperature range. First, the device would adjust the temperature model so that melting peaks coincide across all samples (x-axis calibration). Once aligned, a fluorescence model would be applied to normalize absolute intensity values across channels (y-axis calibration), ensuring consistency in signal interpretation. While this method presents an interesting avenue for improving absolute fluorescence quantification over the full working range, we believe that further development and validation are beyond the scope of this paper.

The precision of temperature control required for detecting SNPs and other genetic variations is critical, and generally, 0.1ºC temperature resolution or better is recommended. Also, errors in temperature measurements at higher temperatures are likely, so calibration curves for the temperature inside the tube measured with a reference thermocouple and temperature measured with the thermistors should have been done at higher temperatures (around denaturation, which is essential for Melt Curve analysis). The peak-to-peak distance while SNP detection can be very close (0.3-0.5ºC approx), which is reported in studies like

- (Harrison LB, Hanson ND. High-Resolution Melting Analysis for Rapid Detection of Sequence Type 131 Escherichia coli. Antimicrob Agents Chemother. 2017 May 24;61(6):e00265-17. doi: 10.1128/AAC.00265-17. PMID: 28416542; PMCID: PMC5444143.) ○ Bruzzone, C.M., Steer, C.J. (2015). High-Resolution Melting Analysis of Single Nucleotide

  Polymorphisms. In: Bugert, P. (eds) Molecular Typing of Blood Cell Antigens. Methods in Molecular Biology, vol 1310. Humana Press, New York, NY. https://doi.org/ 10.1007/978-1-4939-2690-9_2

The ability to detect single nucleotide polymorphisms (SNPs) depends on several factors: the specific mismatch, the buffer, the presence of locked nucleotides or the length of the annealed sequences. Strategies such as using shorter SNP beacons can increase sensitivity, as a single mismatch is more relevant for the overall annealed structure and has a greater impact on melting temperature. For instance, Locked Nucleic Acid (LNA) probes as short as 12 nucleotides have been shown to exhibit significant melting temperature differences—often around 20°C—between perfectly matched targets and those with single mismatches. In this study, we show the detection of mutations ranging from 0 to 4 mismatches on double strands of 90bp annealed in a specific annealing buffer (Fig 2g). The changes on one mismatch were

measured to be about 4-5ºC on these conditions and double checked with a commercial thermocycler.

Future work could explore more stringent tests and calibrate for more precise SNP detection under high-resolution melting conditions to test the limit of detection of the system. A complementary approach would be to adapt biological protocols to be compatible with affordable hardware, simplifying the limits of detection and making the measure compatible with less high-end hardware.

In this study we showcase the ability of the device to perform melting curves. Future work using this feature for a real-world application is out of scope of this paper but would be indeed a really interesting test to move forward. Helpful for detecting emergence of viral variants, environmental screening of the prevalence of SNP conferring AMR, etc.

In any case, temperature precision should not be a concern compared to a commercial thermocycler. In Supplementary Information (Fig. 2), we show that a commercial thermocycler exhibits higher variability across samples when measuring the same melting probes peaks (directly dependent on the reaction temperature). We also attempted measurements using a commercial thermocouple, but in devices with an active heated lid, this became increasingly challenging. When the lid is on, heat transfer down the metallic wire of the sensor is difficult to prevent, causing the measured temperature to become an average between the liquid and the wire temperatures. In a normal setup, without modifying the thermocycler, the wire will inherently be in closer contact with the lid, and it will transmit the temperature downstream faster than the plastic and liquid of the tube. Switching off the lid only provides partial calibration, as in standard reactions the heat from the lid is relevant and affects the metallic holder. We tested thermocouples with both qByte and commercial thermocyclers but were unable to obtain reliable measurements with the lid on. A potential solution we came across was to use very thin AWG wires for the thermocouples to reduce heat conduction down the wire. However, in our attempts, the fragility of these wires made them prone to breaking and difficult to handle in large meaningful numbers, complicating the development of a robust protocol mainly when the number of sensors scale to 96-well plate thermocyclers.

On the other hand, the melting probe assay showcased in Supplementary Information (Fig. 2) consistently produced similar replicable results, regardless of the lid state, and its only requirement is a fluorescence readout. In fact, we found it to be a highly convenient method for using DNA as a biochemical temperature sensor in fluorimeters or any device that requires temperature measurements and has a fluorometric output.

- So, accounting for errors in temperature measurements and temperature resolution is crucial for High-resolution melt curve analysis. However, the authors acknowledge this trade-off between affordability and precision, noting that using cost-effective components and manufacturing methods introduces a higher error level compared to high-end instruments. They suggest potential improvements, including enhancing the optical subsystem for better absolute fluorescence comparison and integrating an additional light channel for ratiometric fluorescence measurements or absorbance readings. Such features could improve the device's accuracy and broaden its applicability in various experimental contexts.

We agree with the reviewer. A further iteration we hope to see in future versions of qByte — as an open-source hardware that can be tweaked now by many — would be to introduce a second wavelength for ratiometric fluorescence measurements or absorbance readings that would render the device more accurate and expand the span of its use cases. This has been now highlighted in the discussion on lines 351-378, that now reads:

Specifically, we identified two main possible future implementations; expanding the measurements from kinetics to absolute fluorescence readings and integrating more readout channels.

In the current design, qByte tracks relative fluorescence changes rather than absolute intensities across its eight channels, which sufficiently supports our demonstrated applications that rely on monitoring signal dynamics over time. However, achieving precise absolute comparisons would be valuable for quantifying absolute concentrations of cells, DNA, or proteins using fluorometric assays, but would require more advanced calibration protocols. As partially explored in Supplementary Data (where we use reference FAM concentrations), these complexities extend beyond a simple intercept or gain correction: minor variations in electrical component performance, metal holder manufacturing, LED and photodiode positioning, and plastic tube properties can significantly affect the intensity of received light, overshadowing absolute signal measurements. One approach to improving calibration without relying on manual fine-tuning is to use previously measured melting probes (see application 1: Nucleic Acid Tests and SD1C) to align fluorescence peaks across a known temperature range. First, the device would adjust the temperature model so that melting peaks coincide across all samples (x-axis calibration). Once aligned, a fluorescence model would be applied to normalize absolute intensity values that correspond to the same intensity across channels (y-axis calibration). While this method presents an interesting avenue for improving absolute fluorescence quantification over the full working range, we believe that further development and validation are beyond the scope of this paper.

The second potential enhancement is adding a second optical channel, either for fluorescence or optical density, to enable ratiometric or absorbance measurements. This would improve signal normalization [44] and enhance the reliability of bacterial studies by referencing fluorescence signals against total cell density [45, 46], which is particularly relevant for applications such as antimicrobial sensitivity testing and, more broadly, systems biology.

The authors have done a great job at standardizing the protocols for the four applications they mentioned and have demonstrated satisfactory results for each. Also, they have made significant efforts in designing and implementing Open-Source Hardware philosophy in developing this device and have given clear instructions to the end user of each step required for manufacturing and assembly of the device. The software protocol is also clearly established to ensure ease of use for the end user. I commend the team on this effort. Kudos!

We truly thank the reviewer for the encouraging words. These were some of the points we more cared about, and we are happy to hear that the publication transmits them to the reader.

Filter Selection and Fluorescent Dyes: Here, commercial LEE filters are used, but for other dyes, it isn't easy to find satisfactory performance using these plastic filters.

- **Background Fluorescence Interference**: Plastic filters can exhibit inherent fluorescence, leading to background signals that interfere with detecting target

fluorescence. A study on reported that conventional color filters, especially those made from certain plastics, can contribute to auto-fluorescence, thereby compromising the sensitivity of fluorescence measurements.

Yamazaki, M., Krishnadasan, S., deMello, A. J., & deMello, J. C. (2012). Non-emissive plastic colour filters for fluorescence detection. Lab on a Chip, 12(21), 4313–4320. https:// doi.org/10.1039/C2LC40718C

Material Stability and Degradation: Plastic filters may degrade over time, especially under prolonged exposure to light used in fluorometric assays. This degradation can alter their optical properties, affecting the accuracy and reliability of fluorescence measurements.

Espí, E., Salmerón, A., & García, J. (2006). Plastic films for agricultural applications. Polymer-Plastics Technology and Engineering, 45(8), 875–883. https://doi.org/10.1177/8756087906064220

We fully agree. For the applications we showcase, these filters offer a good balance between affordability and precision, despite observed background fluorescence. Further work on improved filters could be valuable for more stringent applications. We added this (and the suggested reference) to the relevant section in the Supplementary Information SD1A (Optical signals) to read:

Further improvements to qByte could be achieved by evaluating alternative filters with lower autofluorescence [1], thereby reducing the detection threshold of the device. Care should be taken to monitor potential degradation of the filters' optical properties over time [2]. However, in our tests, no appreciable effects were observed after prolonged use, typically 50 to 100 protocol runs in multiple produced devices (20+). In the event of degradation, qByte allows for easy filter replacement, and given that an entire A1 plastic film costs less than 10$, with only a small portion needed per device, we believe this issue is currently mitigated.

Also, volume variation (well to well) calibration has not been investigated, which can lead to major differences in intensity and lead to errors in Melt curve analysis. This is somewhat mentioned in the discussion section but needs further clarification. Related to previous comment can the authors show sample sizes ranging from 8 uL to 50 uL? This is relevant to many of the applications the authors demonstrate.

Preliminary tests suggest that volume-related variations appear in volumes under 10 µL where the LED light path is close to the liquid-air interface and prone to diffraction. Hence the need to work in volume range of 10 to 40 µL. We now added this to the Hardware Subsystems, Light-Emitting PCB to read (Line 129-133):

The orthogonal placement of the LED in relation to the photodiode decreases background noise. This disposition enables a volumetric working range as low as 10 µL, with an optimal volume of 20 µL or more. Below this threshold, the light hits in the liquid-air interface, which diffracts and prevents coherent and precise measurements.

Minor Comments

- Small changes:
  - should use thermal instead of thermic; thermic represents energy in general, as per Merriam Webster; thermal should be limited to heat. (line number:69)
  - It reaches the photodiode. (line number:94)
- Should mention the wattage of resistors used; otherwise leads to the deterioration of resistors over time and their lifetime. (line number:106)

Many thanks for spotting these errors that were corrected as suggested:

Line 71 and 72 now reads:

...in terms of thermal uniformity and fluorescence kinetics detection.

Line 94 and 95 now reads:

...allowing the light-emitting diode (LED) light to reach the tube chamber and the fluorescence signal to reach the photodiode.

Lines 107 to 109 now reads:

The heated lid and heat-block heater are built on flexible printed circuit boards (PCBs) containing 100Ω SMD resistors (1.5 Watts) that work as the primary heating elements.

Data standardization and interoperability: To enhance data standardization and interoperability for the qByte device, implementing the Real-Time PCR Data Markup Language (RDML) is recommended.

Many thanks for the suggestion. We have modified the repository code and added a button in the analysis tab of the user interface that allows users to download the data in RDML format.

Reviewer 2

The authors have presented a compelling device that can significantly democratize access to biotechnology research. They present application examples with results that show a diversity of successful uses. There are some minor points which might be addressable: a) Lines 312-332 "Specifically, we identified two main possible implementations…." in which the authors describe absolute versus fluorescence change within the samples and so forth. Further in line 324 they describe a second potential improvement. Overall, this paragraph is confusing because the as a reader, I am not sure if you are describing a set of design choices (First vs Second) that you had to take. I am not sure exactly which one you actually took. It left me with more questions about how the device works than answers. Or if you are discussing future considerations. I would restructure that paragraph in a way that it becomes clear and not intermingle theoretical improvements with actual choices. The data speaks for itself and the work is good, this *paragraph* did not increase confidence in your choices. I am sure that was not your intention.

We apologize for the lack of clarity. We have revised it to clearly state two potential future enhancements: first, moving from kinetics to achieving absolute fluorescence measurements through improved calibration protocols; second, integrating a second optical channel. Please refer to the new revised paragraphs spanning lines 351 to 378, now to read:

Specifically, we identified two main possible future implementations; expanding the measurements from kinetics to absolute fluorescence readings and integrating more readout channels.

*In the current design, qByte tracks relative fluorescence changes rather than absolute intensities across its eight channels, which sufficiently supports our demonstrated applications that rely on monitoring signal dynamics over time. However, achieving precise absolute comparisons would be valuable for quantifying absolute concentrations of cells, DNA, or proteins using fluorometric assays, but would require more advanced calibration protocols. As partially explored in Supplementary Data (where we use reference FAM concentrations), these complexities extend beyond a simple intercept or gain correction: minor variations in electri*cal component performance, metal holder manufacturing, LED and photodiode positioning, and plastic tube properties can significantly affect the intensity of received light, overshadowing absolute signal measurements. One approach to improving calibration without relying on manual fine-tuning is to use previously measured melting probes (see application 1: Nucleic Acid Tests and SD1C) to align fluorescence peaks across a known temperature range. First, the device would adjust the temperature model so that melting peaks coincide across all samples (x-axis calibration). Once aligned, a fluorescence model would be applied to normalize absolute intensity values that correspond to the same intensity across channels (y-axis calibration). While this method presents an interesting avenue for improving absolute fluorescence quantification over the fu*ll working range, we believe that further development and validation are beyond the scope of this paper.*

The second potential enhancement is adding a second optical channel, either for fluorescence or optical density, to enable ratiometric or absorbance measurements. This would improve signal normalization [44] and enhance the reliability of bacterial studies by referencing fluorescence signals against total cell density [45, 46], which is particularly relevant for applications such as antimicrobial sensitivity testing and, more broadly, systems biology.

b) Your selection of a wide variety of protocols and applications was refreshing to see beyond a diagnostic approach. Perhaps consider framing the selection in terms of diversity (given the versatile nature of your device) or another theme. Otherwise, the use of TX-TL reactions seems to be slotted in.

To address the reviewer's comment regarding the framing of application diversity, we have made the following modifications:

1. **Framing the diversity of applications** – We have added a sentence in the introduction explicitly stating that the selection of applications was designed to highlight qByte's versatility across different biotechnological fields. This modification can be found on lines 52 to 54. Now it reads:

   The selection of applications was made to highlight the versatility of qByte, spanning different fields—from healthcare-related diagnostics to the democratization of synthetic and systems biology—showcasing its potential beyond a single type of assay.

2. **Clarifying the application title on Genetic Construct Characterisation** – The section previously titled Genetic Construct Characterization has been changed to Genetic Circuit Characterization, as the "Genetic Circuit" keyword better reflects a growing interest in synthetic biology. This change is reflected on line 250.
3. **Providing additional explanation for this particular application selection** – To further clarify the rationale behind including cell-free transcription-translation (TX-TL) reactions, we have added a sentence explaining how this application was chosen to demonstrate qByte's ability to facilitate rapid benchmarking of genetic circuits, reinforcing its broader applicability. This addition can be found on lines 259 to 261 that now reads:

   Aiming to demonstrate qByte's versatility beyond diagnostic applications, we explored its use in combination with TX-TL reactions to perform the characterization of different protein expression constructs.

We appreciate the reviewer's suggestion, which has helped improve the clarity and framing of our manuscript.

c) Given your focus on field deployability: The deployment of the device in Ghana is very well explained, as is the work in the UK (Cambridge), Switzerland (Gaudi Labs), France ("Universit´e Paris Cit´e, INSERM U1284, Center for Research and Interdisciplinarity, F-75006 Paris, France) and in Chile ("Institute for Biological and Medical Engineering, Schools of Engineering, Medicine and Biological Sciences, Pontificia Universidad Catolica de Chile, Santiago, Chile"). This alone is a powerful demonstration of international technology transfer. On the other hand, Line 336 (and its summary in the abstract) detracts from this claim since you omit results (Line 337) in the paper. It

seems to be a distraction. Suggestion: Omit references to field work whose results you are not showing or position it as future work.

To address the reviewer's comment, we have modified the discussion to explicitly state that the fieldwork at Bahia Exploradores in Patagonia is ongoing, ensuring that it is positioned as an active project rather than an unreported result. We believe this is relevant to support the statement that follows on the text on how open-source hardware serves as a tool to establish long-term collaborations. This revision can be found on lines 381–383. Additionally, we have removed references to this work from the abstract to maintain clarity and avoid distractions from the demonstrated results.

In summary, the authors have assembled a formidable interdisciplinary team resulting in a real-world, affordable devices with remarkable international results and implications for democratized biotechnology. This work should be published as soon as possible.

We truly thank the reviewer for the encouraging words.
